# Functional Alignment Philosophy in Total Knee Arthroplasty—Rationale and Technique for the Valgus Morphotype Using an Image Based Robotic Platform and Individualized Planning

**DOI:** 10.3390/jpm13020212

**Published:** 2023-01-26

**Authors:** Jobe Shatrov, Constant Foissey, Moussa Kafelov, Cécile Batailler, Stanislas Gunst, Elvire Servien, Sébastien Lustig

**Affiliations:** 1 Department of Orthopaedics, Croix Rousse Hospital, University of Lyon 1, 69004 Lyon, France; 2 Sydney Orthopaedic Research Institute, Landmark Orthopaedics, St. Leonards, Sydney, NSW 2065, Australia; 3 Claude Bernard Lyon 1 University, IFSTTAR, LBMC UMR_T9406, 69100 Lyon, France; 4 LIBM–EA 7424, Interuniversity Laboratory of Biology of Mobility, Claude Bernard Lyon 1 University, 69622 Lyon, France

**Keywords:** alignment philosophy, knee alignment, functional alignment, total knee arthroplasty, robotically-assisted arthroplasty, valgus morphotype

## Abstract

Functional alignment (FA) is a novel philosophy to deliver a total knee arthroplasty (TKA) that respects individual bony and soft tissue phenotypes within defined limitations. The purpose of this paper is to describe the rationale and technique of FA in the valgus morphotype with the use of an image-based robotic-platform. For the valgus phenotype the principles are personalized pre-operative planning, reconstitution of native coronal alignment without residual varus or valgus of more than 3°, restoration of dynamic sagittal alignment within 5° of neutral, implant sizing to match anatomy, and achievement of defined soft tissue laxity in extension and flexion through implant manipulation within the defined boundaries. An individualized plan is created from pre-operative imaging. Next, a reproducible and quantifiable assessment of soft tissue laxity is performed in extension and flexion. Implant positioning is then manipulated in all three planes if necessary to achieve target gap measurements and a final limb position within a defined coronal and sagittal range. FA is a novel TKA technique that aims to restore constitutional bony alignment and balance the laxity of the soft tissues by placing and sizing implants in a manner that respects variations in individual anatomy and soft tissues within defined limits.

## 1. Introduction

Concepts of alignment in total knee arthroplasty (TKA) are evolving. Mechanical alignment (MA) was first described in the 1980’s as a technique for TKA [1]. Whilst this technique yielded good implant survival rates, dissatisfaction has been reported in 20% of patients [2,3] and half report ongoing symptoms or functional problems following TKA [2]. This combined with the observation that approximately only 50% of the population have a neutral mechanical alignment [4,5] has led to the development of a more individualized approach to TKA.

In pursuit of improving patient function and satisfaction following TKA, alignment philosophies that tailor implant position to patient anatomy have been described [6,7,8]. The term anatomic alignment was coined in 1985 and aimed to restore the joint line obliquity whilst achieving a neutral MA [9]. Kinematic alignment (KA) was later described, aiming to restore the native pre-osteoarthritic knee by resurfacing the joint and accepting the range of laxity produced by natural variation between individual patients [7,10]. This was followed by restricted kinematic alignment (rKA) which aims to reconstitute native alignment but within +/- 3° of a neutral alignment [8].

The arrival of robotic tools has improved quantification and control of implant positioning and balancing targets. Some robotically-assisted platforms offer 3D pre-operative planning with the use of cross-sectional imaging that allow for an assessment of the bony anatomy in all planes to improve implant positioning and sizing [11,12], whilst also offering the ability for precise adjustments to line height, orientation, and intraoperative gaps to be made [13,14]. The use of gaps as a surrogate measure for ligamentous balancing also allows for a quantifiable and reproducible intraoperative surrogate assessment of soft tissue balancing. Achievement of intercompartmental gaps within 2mm of one another (balanced) has been associated with improved pain sores following TKA [15,16,17,18]. The additional control of all these elements with robotic tools has allowed the development of a new philosophy of alignment, termed functional alignment (FA) [19,20,21]. Functional alignment aims to reconstruct 3D constitutional alignment within set boundaries, with a key difference being the adjustment of implant positioning based on consideration of the patient’s soft tissue envelope to achieve gaps that are considered balanced [22]. Its use for the varus morphotype has recently been detailed [23] and was shown to be both bone preserving and more consistent in achieving tibiofemoral balancing and preserving the native trochlea groove compared to KA [23,24]. The technique and rationale for the valgus morphotype is yet to be described.

As the concept of alignment morphotypes has evolved [5,25], so too has the understanding that varus and valgus limbs behave differently and thus require different strategies in TKA [22]. Recently, the valgus morphotype was described as having nine discrete entities based on coronal plane variations in femoro-tibial morphology [26]. TKA for the valgus morphotype has traditionally been considered more demanding [27,28,29,30] due to factors such as extra articular deformity in the tibia [31,32], external tibial rotation (which is coupled with lateral soft tissue contractures that predispose to patellofemoral joint (PFJ) balancing difficulties), medial collateral ligament (MCL) attenuation [31,33], and a significant variability in the morphology of the distal femur [34]. Such variation combined with the aforementioned challenges supports an approach that deals with the specific challenges associated with this morphotype.

FA is an individualized approach that considers the bony constitutional anatomy but also the significant variability of soft tissue laxity between patients. The purpose of this paper is to describe the rationale and our technique of functional alignment for the valgus morphotype using an image-based robotically-assisted platform and preoperative individualized planning.

## 2. Functional Alignment Principles

### 2.1. Individualized Pre-Operative Planning

**Figure 1:**
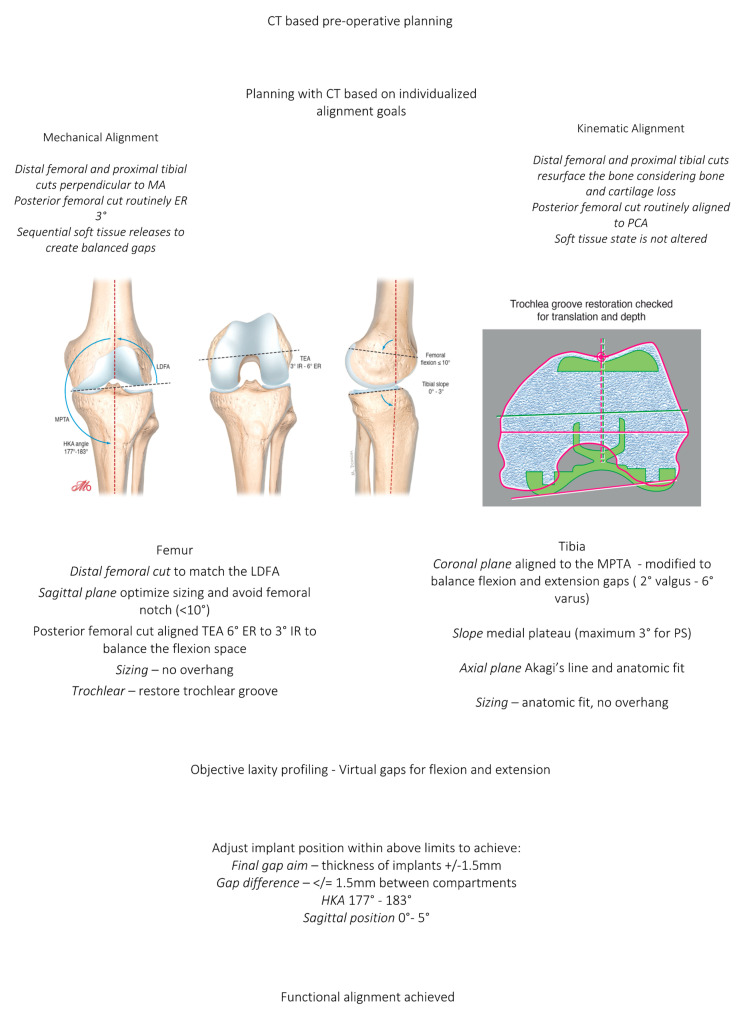
Functional Alignment Workflow for Valgus Morphotype. Functional alignment starts with an individualized plan based on patient anatomy within defined boundaries. Next, the patients soft tissue laxity profile is assessed, and the implant is adjusted within limits to achieve balancing, then the trochlea groove is assessed to ensure the chosen implant position does not compromise the patient’s native sulcus.

Functional alignment for the valgus morphotype begins with the use of 3D imaging to create a personalized plan based on individual anatomy (Figure 1) and within set boundaries (Table 1). The objective of this step is to define the ideal size of the implants as well as anticipate positioning with the aim of restoring the anatomy.

**Figure 2:**
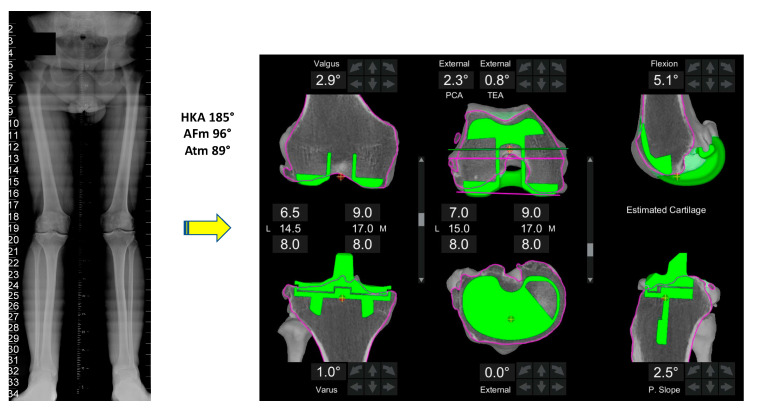
Typical individualized plan for a valgus morphotype knee. A typical FA plan for a valgus morphotype knee is shown. The pre-operative HKA is 5° valgus.

All patients first undergo a standard radiographic evaluation including long leg X-rays to analyze the hip-knee-ankle (HKA) angle, lateral distal femoral angle (LDFA), medial proximal tibial angle (MPTA) and joint line obliquity according to the CPAK classification [35]. 3D imaging (such as a CT scan) is then used to create a personalized plan (Figure 2) within set boundaries (Table 1).

A critical aspect of 3D-based imaging modalities compared to standard 2D modalities is the ability to assess the axial plane, allowing for a more detailed plan of the femoral and tibial rotation before the surgery. This is of paramount importance in the valgus knee due to the wide variations observed in both femoral [36] and tibial rotation [37]. FA creates an individualized plan using the following steps:

#### 2.1.1. Femur

1. Size: posterior referencing with the smallest size that does not overhang mediolaterally, does not notch the anterior femur and avoids overstuffing the patellofemoral joint.
2. Coronal plane: femoral implant positioning is planned according to the LDFA with an upper limit of 6° mechanical valgus in femur. To achieve a final alignment with a maximum valgus of 183°, varus positioning on the tibia may be required unless there is extra-articular varus deformity in either the femur or tibia.
3. Sagittal plane: femoral component is positioned to achieve the best match possible to the concentricity of Blumensaats line in cases without trochlea dysplasia and to avoid femoral notching. This minimizes patellofemoral over or under stuffing.
4. Axial plane: In the valgus morphotype, the posterior lateral femoral condyle is often deficient, making the posterior condylar axis an unreliable landmark for rotation [38]. For this reason, femoral component rotation is planned to balance the flexion space according to the transepicondylar axis (TEA), within a range of 3° internal rotation (IR) to 6° external rotation (ER) to balance the flexion gap. All these adjustments must be made while respecting the alignment with the trochlear groove; care must be taken to assess for trochlea dysplasia which may be present in valgus knees, and if so, recreation of the dysplastic anatomy, such as internally rotating the femur relative to the posterior condylar axis (PCA), should be avoided.
5. Resection depth: For distal femoral resection, the medial femoral condyle represents the reference point. A smaller amount of bone is typically removed from the lateral femoral condyle because of wear and morphological variation that is encountered here. It is 9 mm resection if planned from the medial distal femoral condyle and typically 4-6mm from the lateral femoral condyle. The target of 9 mm resection is based on 7 mm of bone plus 2 mm of cartilage.
6. Medio-lateral positioning: the lateral patella soft tissue structures are typically tight in a valgus knee, and any medialization of the trochlea groove may exacerbate this and potentially cause patellofemoral dysfunction. However, when using a PS femoral component, care must be exercised that lateralization will not lead to a box cut, which will create a fracture of the lateral femoral condyle due to a thin bony remnant in the lateral column.

#### 2.1.2. Tibia

1. Size: The tibia is initially sized with rotation to 0° on the axial view of the CT scan with the aim of having maximal cortical contact with no implant overhang.
2. Coronal plane: tibial implant position is aligned to provide the closest match to the medial proximal tibial axis (MPTA) and balance flexion and extension gaps in a range from 2° of valgus to 6° of varus. In the valgus morphotype, in order to avoid a residual valgus HKA > 3° valgus, the tibia is often cut orthogonal to its mechanical axis. A valgus tibial position more than 2° should be avoided. Valgus tibial positioning is reserved for cases where the deformity is an extra-articular tibial deformity, which in our experience is uncommon.
3. Sagittal plane: tibial implant position is set to match the patient’s native posterior tibial slope with a limit of 3° when using a PS implant and can be modified to balance the flexion gap if necessary. A limit of 10° combined femoral-tibial flexion is allowed.
4. Axial plane: tibial implant is positioned using Akagi’s line. Particular attention should be paid to the avoiding IR of the tibial component, as the valgus morphotype tibia typically falls into ER in deep flexion [36,37] (the position the implant is placed), which promotes the error of internally rotating the tibial baseplate during trials and implantation.
5. Tibial resection: 8 mm resection is planned from the medial tibial plateau (6 mm bone + 2 mm cartilage) based on subchondral bone and an average cartilage depth of 2 mm in the normal knee, to use a 9 mm polyethylene insert. Resection from lateral tibial plateau is, typically less i.e., 4–6mm due to wear, but also to account for the extra articular tibial valgus deformity that often co-exists in the valgus morphotype [31]. This gives a combined planned resection of 17 mm medially, which is the combined thickness of this implant with its thinnest tibial liner.

### 2.2. Coronal Alignment to Aim for Constitutional Alignment within 3° of Neutral

The target coronal alignment guide is set within the limits of 183° (3° valgus) to 177° for a valgus knee. This target is based on the observation that a natural variation in HKA exists from 170° to 183° in native non-osteoarthritic knees [39]. Furthermore, in the valgus morphotype the medial soft tissue structures often become attenuated [30,31,33], which when combined with valgus positioning have been shown to lead to abnormal knee kinematics including excessive anteroposterior (AP) instability [40]. Whilst residual varus in patients undergoing TKA has been shown to not compromise outcomes, residual valgus alignment is less well studied. Lee et al. studied the effect of residual valgus following TKA and concluded that it has no effect on outcomes [41]. However, the data did reveal poorer outcomes with residual valgus beyond 3° and increasing remaining limb valgus resulted in increasing lateral patella tilt and incongruence. This finding is explained by the understanding that residual valgus creates a lateral vector on the patella, but also that valgus knees typically have an externally rotated tibia which laterally translates the tibial tuberosity and acts to laterally tilt and translate the patella as the knee flexes. For these reasons, the residual valgus limb alignment deemed acceptable is a maximum of 3°.

### 2.3. Equal Laxity of Femoro-Tibial Compartments in Flexion and Extension

Soft tissue balancing is a key component of FA. It is also considered one of the most challenging aspects of primary TKA in a valgus knee [27,28,29,30]. With the advent of assistive robotic technology, it has become possible for pre-emptive soft tissue balancing to be incorporated into implant position prior to bone cuts being performed. This has recently been shown to reduce the amount of bone resected as well as reducing the need for soft tissue releases in TKA for the varus morphotype [23].

The aim is to achieve flexion and extension gaps goals in the medial and lateral compartments that are within 1.5 mm of the global thickness of the implant (which is 17.5 mm when a 9 mm polyethylene insert is used). This limit is chosen to have residual gaps of less than 2 mm in both tibiofemoral compartments. In simple terms, this means finishing with gaps in both tibiofemoral compartments (medial/lateral) that are equal to or less than 1.5 mm of each other. Additionally, the final gaps in full extension and 90° flexion should not be more than 2 mm from the global implant thickness (i.e., 19.5 mm for a 17.5 mm thickness).

**Figure 3:**
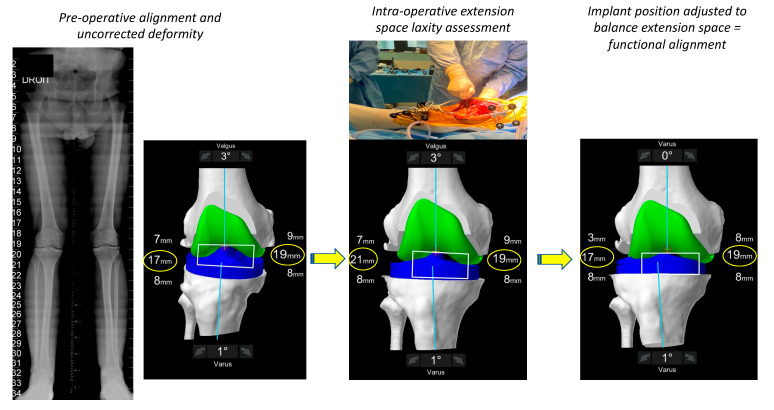
Intra-operative assessment of the extension space. The limb is placed in a corrected position and the robot ‘captures’ the pose. The plan will deliver extension gaps of 18 mm laterally and 17 mm medially. In order to achieve balanced compartments, the plan is modified in this case by decreasing the femoral valgus.

**Figure 4:**
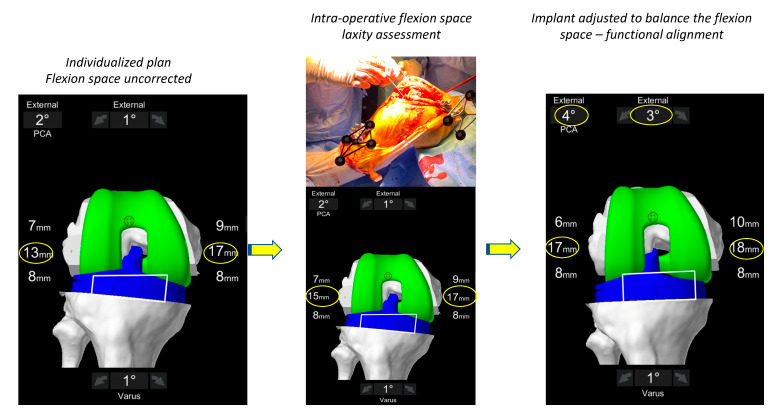
Intraoperative assessment of the flexion space. The flexion space is assessed using sized spacer spoons until the corrected position is achieved. The personalized plan will deliver a lateral space of 17 mm and a medial space of 18 mm. In order to balance the flexion space, the implant is externally rotated until the compartments are balanced.

During the assessment of the soft tissue laxity assessment, the surgeon places the lower limb in the ‘corrected’ position in both flexion and extension (Figure 3 and Figure 4). Multiple methods exist for assessing soft tissue laxity including soft tissue tensiometers, manual correction by the surgeon or use of spacer blocks or spoons. Regardless of the chosen method, a major point of contention requiring further research is how much force to place on the limb when measuring the patient’s soft tissue envelope. Our technique utilizes spacer spoons that compensate for cartilage and bone wear and has been described previously [22]. Assessment of the medial compartment laxity is technically easier, as this space is rigidly constrained by the medial collateral through an arc of flexion [42] meaning that if the medial structures are not attenuated the medial compartment in its normal state should have very little residual gap, and during testing, should be stressed only until the medial soft tissue envelope first becomes taught. However, the lateral side is more difficult, being more constrained in extension but having some laxity in flexion. However, the complexity of measuring the lateral compartment laxity lies in the fact that it is also constrained by structures controlled by muscles (tensor facia lata, popliteus and biceps femoris) whose contribution to laxity cannot be assessed intra-operatively.

**Figure 5:**
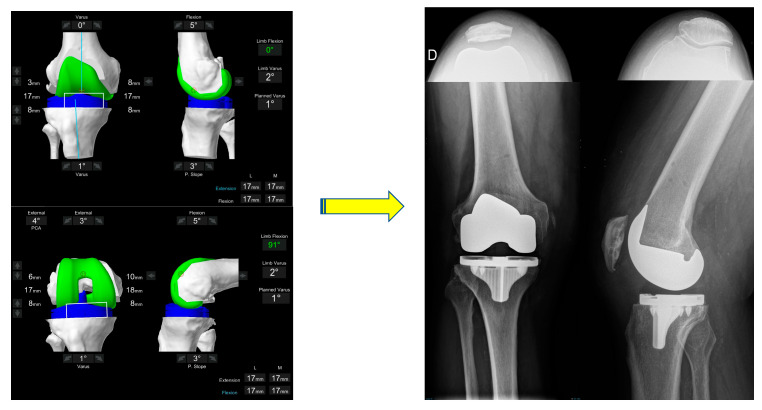
The final intra-operative position. Limb alignment, resections depth and tibiofemoral gaps in flexion and extension are shown. The achieved coronal alignment is 178°, or 2° varus and the sagittal alignment is 0° of flexion. The final gaps are 17 and 18mm, respectively. The post-operative X-ray at 3 months follow-up of the same patient is shown. The patella is well centred on the skyline X-ray.

The anticipated effect of the pre-operative plan on the gaps at 0° and 90° is demonstrated and the surgeon determines if either soft tissue releases or implant adjustments are required (Figure 3 and Figure 4). In FA, implant adjustments within defined boundaries are performed. If the residual alignment falls outside the target range of <3° valgus, selective soft tissue releases can be performed (posterolateral corner and ilio-tibial band); however, in our experience to date with FA this is rarely required. Final gaps are assessed in both full extension and 90° flexion once implantation has occurred (Figure 5).

### 2.4. Final Limb Sagittal Alignment to Achieve Full Extension

Sagittal limb alignment refers to the position of the knee as measured by the robotic system with only the force of gravity, i.e. when the foot is held off the table with the patella in the reduced position. One of the surgical goals in FA is correction of the sagittal deformity (0° extension under gravity). The precise effect of residual sagittal limb alignment in TKA remains unclear. Whilst satisfaction and PROM’s appear to be improved when a fixed-flexion-deformity is eliminated [43], this effect does not appear to be observed when the residual position is less than 5 degrees from neutral [44]. We have recently demonstrated that a residual recurvatum deformity of 5 degrees can be well tolerated at a 5-year follow-up using a PS implant in patients who initially had a recurvatum deformity pre-operatively [45]. For this reason, we aim for the final limb alignment to be no more than 5° from neutral following implantation of final components.

## 3. Discussion

**Figure 6:**
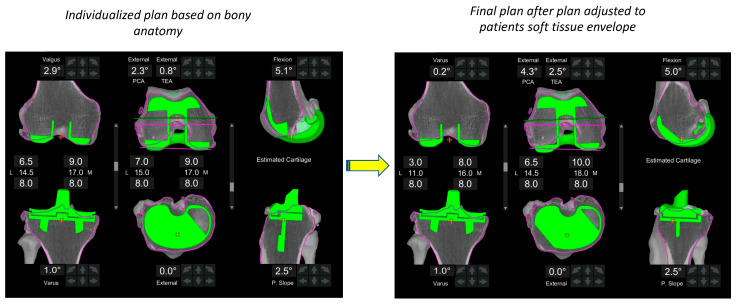
Functional alignment. The major difference between the FA and previous philosophies is the modification of alignment based on a combination of constitutional bony anatomy and soft-tissue laxity assessment. In this example, the plan (left image) is adapted within defined boundaries (right image) based on the patient’s soft tissue envelope in order to achieve balance targets.

The aim of this paper was to describe the FA philosophy in TKA for the valgus morphotype using a robotic platform. FA is a philosophy that utilizes image-based robotically-assisted technology to perform TKA that aims to recreate patients’ constitutional alignment within defined boundaries and makes adjustments to the implant positions being made based on a quantifiable soft tissue laxity profile (Figure 6). Finally, FA considers the behavior of varus and valgus morphotypes to be significantly different requiring a different approach for each.

### 3.1. Morphotypes

Understanding of limb alignment has developed significantly in the last decade. The initial belief that normal limb alignment is always a straight neutral mechanical axis [1] has changed. In 2012, Bellemans et al. noted that in a non-arthritic population only 32% of males and 17% of females had a neutral mechanical axis [4]. This was followed by similar findings by Hirschmann et al. [5], then Macdessi et al. [35] who in addition to these findings also both described concepts of defining and grouping patients based on joint line obliquity. As the concept of alignment morphotypes has evolved [5,25], the understanding that varus and valgus limbs behave differently and thus may require alternate strategies in TKA has also emerged [22]. Recently, the valgus morphotype was described as having nine discrete entities depending on the origin of the deformity (intra-articular and/or extra-articular, femur and/or tibia) [26]. Such variation demands a more individualized approach to TKA.

### 3.2. Soft Tissue Balancing

The major difference of the FA to previous philosophies is the modification of alignment based on a combination of constitutional bony anatomy and soft-tissue laxity assessment (Figure 6). Instability after knee arthroplasty is a cause for failure [15,46], particularly in younger patients [47], and pain and satisfaction scores are improved when target ranges of tibio-femoral gaps are achieved intra-operatively [17,48,49,50]. Defining balance remains difficult and may differ based on prosthesis design i.e. Medial pivot versus PS, CR constrained designs, or single versus multi-radius curvature femoral components. Variation in laxity of the collateral ligaments [51] in non-arthritic knees, between genders [52] and in flexion versus extension [51], has been observed. In addition to this, in the valgus knee, attenuation of structures on the deformities convex medial side may occur, with reciprocal shortening on the concave lateral side [33]. Aunan et al. reported a medial laxity greater than 2 mm in extension and 3 mm in flexion was associated with poorer functional outcomes more than 1 year after TKA in neutral and valgus aligned TKA [53]. Failure to account for the soft tissue variability between individuals and the changes characteristic of the valgus morphotype may lead to imbalance, residual deformity, and early failure.

Debate regarding gap targets in TKA is ongoing. Whilst the wide variation in laxity in varus aligned knees has been described [51], less data is available regarding the valgus morphotype. Important differences exist between the native and prosthetic knee, with both cruciate ligaments being sacrificed in PS TKA prosthesis, for example. Resection of the PCL increases flexion laxity and can create a medial-lateral compartment mismatch if not adjusted appropriately [54]. Several definitions of balancing have been described, with many accepting some asymmetry between the medial and lateral compartments [55,56,57,58,59,60]. It is unclear if native balancing targets should be replicated in prosthetic designs that are cruciate deficient, and the optimal balancing targets for varus and valgus morphotypes in the tibiofemoral compartments remains elusive. Regardless, the additional data provided by robotic technology will allow this question to be examined more closely in the near future.

### 3.3. Coronal Alignment

Coronal positioning of the femoral implant is heavily influenced by alignment choice. The defined target safe zones for final HKA are not yet resolved. Whilst limited residual varus alignment in TKA has been shown to be a safe approach [61,62], little data is available regarding the effect of residual valgus alignment. Lee et al. demonstrated that beyond 3° of residual valgus alignment, patella femoral incongruence and lateral tilt were observed [41]. Concerningly, patellofemoral complications are also the most common reason for revision following KA TKA and typically occur in the valgus morphotype [63,64,65]. In the current FA technique for the valgus morphotype, a residual limit of maximum 3° valgus is therefore chosen. However, in patients with increased valgus in the anatomical axis of the femur, to avoid excessive valgus, a typically asymmetric resection is then required in the distal femoral cut, with less bone being remove from the lateral femoral condyle, resulting in it being lengthened. Functional leg lengthening has been shown to occur in patients when correcting for valgus deformity in TKA, with a significant amount of this occurring in the femur [66]. The clinical consequences of this remain unknown and could be an avenue for future research. Further research is also required to understand the effect of residual valgus on knee kinematics and its clinical consequences.

### 3.4. Femoral Rotation

Functional alignment uses the TEA defined on the pre-operative CT scan as the landmark for rotation of the femoral component. Talbot et al. demonstrated that the anatomical TEA axis has a narrower range than the PCA [67]. The implications being that following the PCA for rotational alignment, as is the case in KA, will lead to an excessively internally rotated femoral component. This is particularly relevant in the valgus morphotype where lateral condyle bone deficiency is encountered in at least 25% of cases [26], making the PCA an unreliable landmark. Recently it was demonstrated that using the PCA as reference for femoral component position, as is the case in KA, led to more than 20% of TKA’s having a femoral component that was more than 3° internally rotated relative to the TEA [24]. Use of the TEA as a landmark for rotation has in the past also been fraught with poor inter- and intra-observer reliability [68]; however, with the addition of CT landmarking in image-based robotic platforms, this issue is dealt with and the TEA becomes a more reproducible landmark for axial component positioning.

Defining boundaries for rotational alignment of the femoral component in the valgus knee is complex. It is well accepted that femoral rotation affects patellofemoral kinematics, but also plays an important role in flexion stability and tibiofemoral kinematics [68]. Functional alignment boundaries for femoral rotation are set at 6° ER to 3 IR ° in relation to the TEA. The rationale for this boundary is based on data from Berger et al. [69] who reported a direct relationship between the amount of IR of the femoral component and patellofemoral complications. Internal rotation of 1–4° was associated with lateral patellar tracking and tilt, and IR beyond 5° was associated with patellar subluxation, dislocation, and patella component failure. These limits have recently been challenged by Clatworthy et al. who reported no deleterious outcomes with the use of a patient specific protocol of femoral rotation that varied from 7° of IR to 8°of ER. However, this was in relation to the PCA using an imageless navigation system and future research is required to understand safe-zone boundaries as well the tolerance of various implant designs to extreme positioning.

The concept of morphotypes and an individualized approach to TKA are relatively new. Owing to this, understanding is likely to continue to evolve as more research comes to light. Ultimately this, combined with new developments in robotic technology and implant design will likely see the FA philosophy also evolve. However, for the moment, based on our current experience, understanding, and published data, we present our approach to the valgus morphotype in TKA, from Lyon, France.

### 3.5. Future Study and Weakness’s

Several questions remain unanswered. First, this technique assumes that the target for balancing in extension, and then at 90° flexion, results in a balanced knee throughout a range of motion which makes sense with a single radius implant but remains untested. Furthermore, this technique aims for similar gaps in the medial and lateral compartment, which differs from that of native knees. However, the question of ideal gap targets in prosthesis that are cruciate deficient remains unanswered. Furthermore, whilst the laxity variation of the varus morphotype has been described, less is known about the valgus morphotype, and it remains to be seen whether target gaps should be tailored to the individual patient’s arthritic soft tissue envelope or be altered to match the thickness of the implant, as is the current approach in FA. Finally, concern regarding safe limits for implant positioning remain, with thresholds being vaguely defined and with little evidence to support rigid cut-offs. Ultimately, the answer to this question will only be realized with long-term follow-up data which is yet to come.

## 4. Conclusions

Functional alignment is a novel knee arthroplasty technique that aims to restore constitutional bony alignment and balance the laxity of the soft tissues by placing and sizing implants in a manner that respects variations in individual anatomy and soft tissues within defined limits. This paper presents how this personalized approach can be achieved for the valgus morphotype.

## Figures and Tables

**Table 1:** Functional Alignment Philosophy Protocol Guidelines for the valgus morphotype.

Parameter	Target
Final coronal alignment (HKA)	177°–183°
Final sagittal alignment with gravity only	0° +/− 5°
Femur	
Varus/valgus *	3° varus to 6° valgus
Flexion *	0°–10°
Transepicondylar axis (TEA)	3° IR–6° ER
Tibia Varus/valgus * Slope * Rotation	2° valgus to 6° varus 0°–3° (depends if CR/CS or PS) Manual Combination of Akagi’s line, anatomic fit and floating method
Implant sizing	Femur—matched to curvature of distal femoral radius to avoid notching and medial-lateral condyles to avoid any overhang or over-stuffing Tibia—maximal cortical contact with correct rotation with no overhang Downsized if there is any conflict
Balancing	Gaps to match the global thickness of the implant at: - 0° extension - 90° flexion Maximum gap difference 1mm between medial and lateral compartments with a slight lateral laxity acceptable

* Combined values between tibia and femur more important than isolated values. Individual manufacturing guides may vary between implants.

## Data Availability

Not applicable.
